# The bromodomain and extra-terminal domain degrader MZ1 exhibits preclinical anti-tumoral activity in diffuse large B-cell lymphoma of the activated B cell-like type

**DOI:** 10.37349/etat.2021.00065

**Published:** 2021-12-31

**Authors:** Chiara Tarantelli, Eleonora Cannas, Hillarie Ekeh, Carmelo Moscatello, Eugenio Gaudio, Luciano Cascione, Sara Napoli, Cesare Rech, Andrea Testa, Chiara Maniaci, Andrea Rinaldi, Emanuele Zucca, Anastasios Stathis, Alessio Ciulli, Francesco Bertoni

**Affiliations:** 1 Institute of Oncology Research, Faculty of Biomedical Sciences, USI, 6500 Bellinzona, Switzerland; 2 Department of Medical, Oral and Biotechnological Sciences, “G. d’Annunzio” University of Chieti-Pescara, I-66100 Chieti, Italy; 3 SIB Swiss Institute of Bioinformatics, 1000 Lausanne, Switzerland; 4 Division of Biological Chemistry and Drug Discovery, School of Life Sciences, University of Dundee, Dundee, DD1 5EH, Scotland, UK; 5 Oncology Institute of Southern Switzerland, Ente Ospedaliero Cantonale, 6500 Bellinzona, Switzerland; 6 Faculty of Biomedical Sciences, USI, 6900 Lugano, Switzerland; University of Southampton, UK

**Keywords:** BET, bromodomain, bromodomain-containing protein 4, lymphoma, diffuse large B-cell lymphoma, proteolysis-targeting chimeras, immuno-oncology, epigenetics

## Abstract

**Aim:**

Bromodomain and extra-terminal domain (BET) proteins are epigenetic readers that play a fundamental role in transcription regulation. Preclinical and early clinical evidence sustain BET targeting as an anti-cancer approach. BET degraders are chimeric compounds comprising of a BET inhibitor, which allows the binding to BET bromodomains, linked to a small molecule, binder for an E3 ubiquitin ligase complex, triggering BET proteins degradation via the proteasome. These degraders, called proteolysis-targeting chimeras (PROTACs), can exhibit greater target specificity compared to BET inhibitors and overcome some of their limitations, such as the upregulation of the BET proteins themselves. Here are presented data on the anti-tumor activity and the mechanism of action of the BET degrader MZ1 in diffuse large B cell lymphoma (DLBCL) of the activated B-cell like (ABC, ABC DLBCL), using a BET inhibitor as a comparison.

**Methods:**

Established lymphoma cell lines were exposed for 72 h to increasing doses of the compounds. Cell proliferation was evaluated by using an 3-(4,5-dimethylthiazolyl-2)-2,5-diphenyltetrazoliumbromide (MTT) assay. Fluorescent-Activated Cell Sorter (FACS) analysis was performed to measure apoptotic activation and RNA sequencing (RNA-Seq) to study the transcriptional changes induced by the compounds.

**Results:**

MZ1, and not its negative control epimer cisMZ1, was very active with a median half maximal inhibitory concentration (IC_50_) of 49 nmol/L. MZ1 was more *in vitro* active than the BET inhibitor birabresib (OTX015). Importantly, MZ1 induced cell death in all the ABC DLBCL cell lines, while the BET inhibitor was cytotoxic only in a fraction of them. BET degrader and inhibitor shared partially similar changes at transcriptome level but the MZ1 effect was stronger and overlapped with that caused cyclin-dependent kinase 9 (CDK9) inhibition.

**Conclusions:**

The BET degrader MZ1 had strong cytotoxic activity in all the ABC DLBCL cell lines that were tested, and, at least *in vitro*, it elicited more profound effects than BET inhibitors, and encourages further investigations.

## Introduction

Diffuse large B cell lymphoma (DLBCL) comprises two biologically distinct entities that are referred to as germinal center B-cell (GCB) and activated B-cell (ABC) subtype [1–3]. These two subtypes of DLBCL have different survival patterns with a substantially worse outcome for ABC than GCB DLBCL patients when treated with standard chemoimmunotherapy [1]. ABC and GCB DLBCL show different gene expression profiles and display specific oncogenic pathway perturbations, such as a constitutively active nuclear factor kappa B (NF-κB) and Janus tyrosine kinase (JAK)/ signal transducer and activator of transcription 3 (STAT3) signaling in the ABC subtype [2, 3]. More recent studies have further defined the subclassification of DLBCL, identifying a series of genetically defined subclusters including the largely overlapping MCD and cluster 5 (C5), exclusively comprising ABC DLBCL and associated with dismal survival [4–6].

Preclinical [7–10] and early clinical [11, 12] data show that ABC DLBCL can be sensitive to pharmacological inhibition of the proteins belonging to the bromodomain and extra-terminal domain (BET) family. BET proteins are epigenetic readers that play a fundamental role in transcription regulation [13–15]. They include the ubiquitously expressed bromodomain-containing protein 2 (BRD2), BRD3, and BRD4 and the testis-restricted bromodomain testis-specific protein (BRDT). BET proteins share an extra-terminal domain (ET), conserved N-terminal bromodomains [BD; first bromodomain (BD1) and second bromodomain (BD2)], and, only in BRD4 and BRDT, a C-terminal domain. BET proteins are localized at acetylated lysine residues on promoter regions and enhancers of active genes, where they recruit transcriptional regulatory complexes to acetylated chromatin and regulate transcription initiation and elongation. The precise function of each BET protein is not well defined, and they present only partial overlaps among them in terms of interacting proteins, an indication that they likely have a different function in different cellular contexts [16–19]. BET inhibitors block the binding of BET proteins to acetylated histones, and since they do this targeting the BD conserved regions, the majority of them are pan-BET inhibitors, even if they present differences in their preferential binding to BD1, BD2, or both [15]. BET inhibitors repress the transcription of active genes, as key oncogenes, and anti-apoptotic proteins [20, 21]. The inhibition of transcription is particularly strong for those transcripts with regulatory regions with a high enrichment for the binding of transcriptional coactivators, such as BRD4. Among genes that are inhibited by BET inhibitors, there are genes essential for ABC DLBCL cells [7–9, 22]. BRD4 can also regulate the NF-κB signaling also by directly binding to the RelA protein [23]. Finally, Eμ-BRD2 transgenic mice over-expressing BRD2 in the B-cell compartment, develop aggressive B-cell leukemias and lymphomas resembling DLBCL with an ABC phenotype [24, 25]. Although BET inhibitors are active in all ABC DLBCL cell lines, their activity is largely cytostatic with a strong induction of apoptosis limited to cell lines bearing somatic mutations in essential genes such as myeloid differentiation factor 88 (*MYD88*) and cluster of differentiation 79B (*CD79B*) [9, 10], representative of the genetically defined subclusters MCD/C5 [4–6].

The clinical results so far obtained with BET inhibitors have been rather limited [11, 12, 15, 26– 33]. One of the main factors that limit the anti-tumor activity of BET inhibitors is the frequently induced upregulation of BET proteins themselves [15, 34–37]. This limitation is overcome by the use of BET degraders, compounds based on the concept of proteolysis-targeting chimeras (PROTACs) and consisting in a BET inhibitor moiety, linked to an additional small molecule which recruits an E3 ubiquitin ligase complex, triggering BET protein ubiquitination and proteasome-mediated degradation [38–40]. BET degraders with different BET-binding motives and E3 recruiters have been generated [15, 41]. MZ1 is a potent BET degrader composed of the BET inhibitor JQ1 for its BET BD binding and a ligand for the E3 ubiquitin ligase von Hippel-Lindau (VHL) for proteasomal degradation [42]. Here, we present data on the anti-tumor activity of MZ1 in ABC DLBCL *in vitro* and *in vivo* models.

## Materials and methods

### Cell lines and molecules

Seven established ABC DLBCL cell lines (HBL1, OCI-LY-10, OCI-LY-3, RI-1, SU-DHL-2, TMD8, and U2932) were all validated for their cell identity by short tandem repeat (STR) DNA fingerprinting (Promega GenePrint 10 System kit) [43]. All media were supplemented with fetal bovine serum (FBS; 10%), penicillin-streptomycin-neomycin (~5,000 units penicillin, 5 mg streptomycin and 10 mg neomycin/mL, Sigma), and *L*-glutamine (1%). MZ1 and cisMZ1 were synthesized following a previously established synthetic route [42], while birabresib (OTX015) was purchased (Selleck Chemicals, Houston, TX, USA). All the molecules were dissolved in dimethyl sulfoxide (DMSO) at 10 mmol/L stock solution.

### Cell proliferation assay

The antiproliferative activity of MZ1, cisMZ1, and birabresib as a single agent was assessed as previously described [9]. The compounds were serially diluted in the appropriate tissue culture medium, at a range of 0.95–1,000 nmol/L, and added to cells (two biological replicates) at a maximum of 0.1% DMSO (v/v). Cells were incubated for 72 h at 37°C, 5% CO_2_. DMSO alone was added to negative control (untreated) cells. Wells containing medium only were included on each plate and served as blanks for absorbance readings. 3-(4,5-dimethylthiazolyl-2)-2,5-diphenyltetrazoliumbromide (MTT, Sigma-Aldrich Chemie GmbH, Buchs, Switzerland) was prepared as a 5 mg/mL stock in phosphate buffered saline (PBS) and filter-sterilized. MTT solution (20 μL) was added to each well and tissue culture plates were incubated at 37°C for 4 h. Cells were then lysed with 25% sodium dodecyl sulfate (SDS) lysis buffer (50 μL) and absorbance was read at 570 nm using Cytation3 plate reader (Biotek Instruments, VT, USA). The doses corresponding to the half maximal inhibitory concentration (IC_50 _) and area under the curve (AUC) were estimated by fitting a sigmoidal model through the dose-response curve using the R statistical package (www.r-project.org).

### Immunoblotting analysis

Immunoblots of cell line protein extracts were performed as previously described [44]. Proteins were extracted in an appropriate volume of mammalian protein extraction reagent (M-PER) lysis buffer added with protease and phosphatase inhibitors (Thermo Fisher Scientific, MA, USA). The protein content was determined using the bicinchoninic acid (BCA) protein assay (Pierce Chemical Co). Lysates were fractionated by 8% SDS-polyacrylamide gel electrophoresis (SDS-PAGE). Membranes were incubated with the primary antibodies overnight, followed by the appropriate horseradish peroxidase-conjugated anti-mouse or anti-rabbit secondary antibodies (Amersham Life Science) for 1 h. Enhanced chemiluminescence detection was then done following the manufacturer’s instructions (Amersham Life Science). Equal loading of samples was confirmed by probing for glyceraldehyde-3-phosphate dehydrogenase (GAPDH) or actin. Used antibodies are pSTAT3-Tyr705 (CST 9131), STAT3 (CST 9139), MYC (CST D3N8F), (Cell Signaling Technology CST, Danvers, MA, USA); BRD4 (SC-48772), BRD3 (SC-81202), BRD2 (SC-81825), (Santa Cruz Biotechnology, Santa Cruz, CA, USA) and GAPDH (FF26A, Centro Nacional De Investigaciones Oncologicas CNIO, Madrid, Spain).

### Cell cycle and apoptosis assay

Cell cycle was performed as previously described [9]. Briefly, 1 × 10^6^ cells/mL were cultured for 72 h, harvested and washed twice in PBS with 1% FBS, permeabilized with 70% ethanol, and resuspended in PBS with propidium iodide (PI; 1 mg/mL, Sigma-Aldrich) and ribonuclease (RNase). After 20 min of incubation, cells were analyzed using flow cytometer [BD Fluorescent-Activated Cell Sorter (FACS) Canto I]. The analysis of the percentage of cell death the percentages of cells in G1, S, and G2/M phases of the cell cycle was determined using the Watson Pragmatic model and the FlowJo software (TreeStar Inc., Ashland, OR, USA). Cells (3 × 10^5^ cells/mL) were cultured for 72 h, and the percentage of viable and apoptotic cells [Annexin V positive/7-Aminoactinomycin D (7AAD) negative and Annexin V positive/7AAD positive] was determined by double staining the cells with Annexin V-FITC/7AAD.

### *In vivo* experiment

Mice maintenance and animal experiments were performed under the institutional guidelines established for the Animal Facility and with study protocols approved by the local Cantonal Veterinary Authority (license TI-23/2015). NOD.CB17-*Prkdcscid*/NCrHsd (NOD-SCID) mice were obtained from The Harlan Laboratory (S. Pietro al Natisone, Udine, IT). Xenografts were established by injecting TMD8 lymphoma cells (15 × 10^6^ cells/mouse, 200 μL of PBS) into the left flanks of female NOD-SCID mice (6 weeks of age, approximately 20 g of body weight). Compounds were dissolved in 25% hydroxypropyl-beta-cyclodextrin (HP-β-CD), and adjusted pH 6.0 in an application volume of 4 ml/kg, as previously done [45]. Tumor size was measured on regular basis and until tumors reached around 5 mm in diameter (100 mm^3^). The body condition scoring was used to assess mice’s health status [46]. Differences in tumor volumes [(length × width × width)/2] were calculated using the Wilcoxon rank-sum test (Stata/SE 12.1 for Mac, Stata Corporation, College Station, TX). The *P*-value (*P*) for significance was < 0.05.

### Transcriptome profiling

RNA was extracted and processed for RNA sequencing (RNA-Seq; stranded, single-ended 75 bp-long sequencing reads) using the NEBNext Ultra Directional RNA Library Prep Kit for Illumina (New England BioLabs Inc., Ipswich, MA, USA) on a NextSeq 500 (Illumina, San Diego, CA, USA) as previously described [47]. Data mining was performed as previously described [48]. Differentially expressed transcripts were defined as those with an average normalized log expression [counts per million reads mapped (cpm)] of at least 2, presenting an absolute |logFC| > 1 and Benjamini-Hochberg (BH) multiple tests corrected *P* value [false discovery rate (FDR)] < 0.05. Functional annotation was done using Gene Set Enrichment Analysis (GSEA) on fold-change pre-ranked lists of genes considered differentially expressed. Genesets from the MSigDB collection (hallmark, c2cpg, c2cp, c6) [49], SignatureDB [50] and from different experimental conditions [9, 10, 44], applying as thresholds *P* < 0.05 and FDR values < 0.1. Omics playground tool [51] was used to compare the expression profiles against the L1000 drug expression database [52]. Expression data are available at the National Center for Biotechnology Information Gene Expression Omnibus (GEO number, GSE152497; http://www.ncbi.nlm.nih.gov/geo) database. The transcriptome study was performed in the context of a larger work assessing also other agents; hence, the DMSO and the birabresib raw data have been previously reported [53].

## Results

### The BET degrader MZ1 has *in vitro* cytotoxic activity in ABC DLBCL

**Figure 1.**
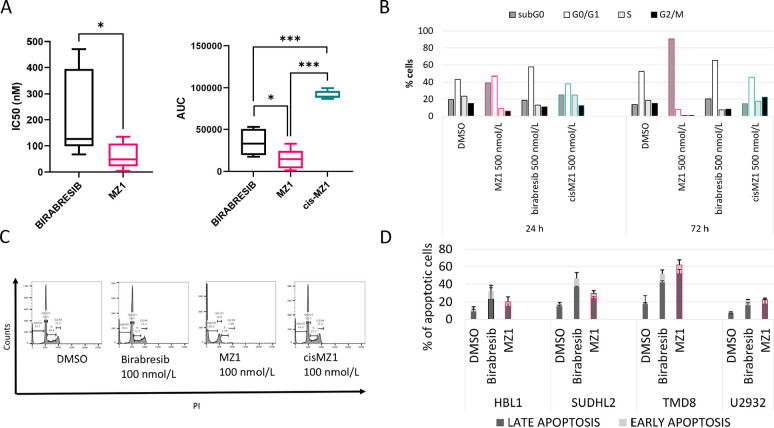
Activity of MZ1, birabresib and cisMZ1 in ABC DLBCL cell lines. A. Seven ABC DLBCL lymphoma cell lines were exposed to increasing concentrations of MZ1, birabresib or cisMZ1 for 72 h. IC_50_ and AUC were calculated and showed in the in the box plots; B. cell cycle effects observed after 24 h and 72 h of treatment of OCI-LY-10 cells with birabresib, MZ1 and cisMZ1 (500 nmol/L); C. representative plots obtained after 72 h of treatment at 500 nmol/L; D. apoptotic effect of the BET degrader MZ1 and birabresib in four ABC DLBCL cell lines tested at their IC_50_s, as in Table 1. Early and late apoptotic effect for 72 h was evaluated. DMSO *vs.* birabresib, *P* < 0.05. DMSO treatment was used as a control. MZ1, cisMZ1 and birabresib were dissolved in DMSO and maximum 0.1% DMSO was used for treatments. The Mann-Whitney *U* test was used to compare groups. ^*^ *P* < 0.05; ^***^ *P* < 0.001

Seven cell lines derived from ABC DLBCL were exposed for 72 h to the BET degrader MZ1, its negative control epimer cisMZ1 and, as a comparison, the pan-BET inhibitor birabresib which has shown early clinical activity [12, 27]. MZ1 was very active with a median IC_50_ of 49 nmol/L [95% confidence interval (CI), 10.16–126.76 nmol/L] and a median AUC of 14,789 (95% CI, 1,302–32,997) (Figure 1A, Table 1, Figure S1). The BET degrader was more active than the BET inhibitor, which presented a median IC_50_ of 126 nmol/L (95% CI, 77.83–446.64 nmol/L; *P* = 0.024) and a median AUC of 33,067 (95% CI, 17,690–53,025; birabresib *vs.* MZ1, *P* = 0.012). No activity was seen with cisMZ1, which showed a median AUC of 89,072 (95% CI, 86,715–99,547; cisMZ1 *vs.* birabresib, *P* < 0.001; cisMZ1 *vs.* MZ1, *P* < 0.001).

**Table 1.** Activity of MZ1, birabresib and cisMZ1 in ABC DLBCL cell lines

**Cell line**	**MZ1 (nmol/L)**		**Birabresib (nmol/L)**		**CisMZ1 (nmol/L)**	
	**IC_50_**	**AUC**	**IC_50_**	**AUC**	**IC_50_**	**AUC**
HBL1	61.68	17,036	292.38	43,057	> 2,000	89,050
OCI-LY-10	4.39	1,302	125.91	33,067	> 2,000	94,936
OCI-LY-3	22.76	3,934	98.35	26,973	> 2,000	89,072
RI-1	134.88	32,997	394.42	50,586	> 2,000	86,715
SU-DHL-2	48.95	14,789	68.42	17,690	> 2,000	88,269
TMD8	39.10	6,847	106.92	19,826	> 2,000	99,547
U2932	109.03	24,749	470.57	53,025	> 2,000	96,221

Median IC_50_	48.95		125.91		n.a.	
95% CI	10.16–126.76		77.83–446.64		n.a.	

Median AUC		14,789		33,067		89,072
95% CI		1,302–32,997		17,690–53,025		86,715–99,547

Seven ABC DLBCL lymphoma cell lines were exposed to increasing concentrations of MZ1, birabresib or cisMZ1 for 72 h. Table shows the IC_50_ and AUC values for each cell line, 95% CI of median IC_50_ and AUC for each drug. n.a.: not avalable

MZ1 (500 nmol/L) induced an accumulation of OCI-LY-10 cells in the subG0 phase already after 24 h of treatment, while birabresib (500 nmol/L) induced G0/G1 accumulation in agreement with what previously reported (Figure 1B–C) [9]. Both effects were more pronounced at 72 h. No changes in the cell cycle occurred with cisMZ1. MZ1 tested at IC_50_ concentration in four ABC DLBCL cell lines for 72 h showed that MZ1 induces cell death in only 1/4 cell lines more than birabresib (TMD8), while the latter is inducing more cell death in 2/4 cells (HBL1, SUDHL2) (Figure 1D).

### The BET degrader MZ1 strongly suppresses BRD4 and MYC protein levels

**Figure 2.**
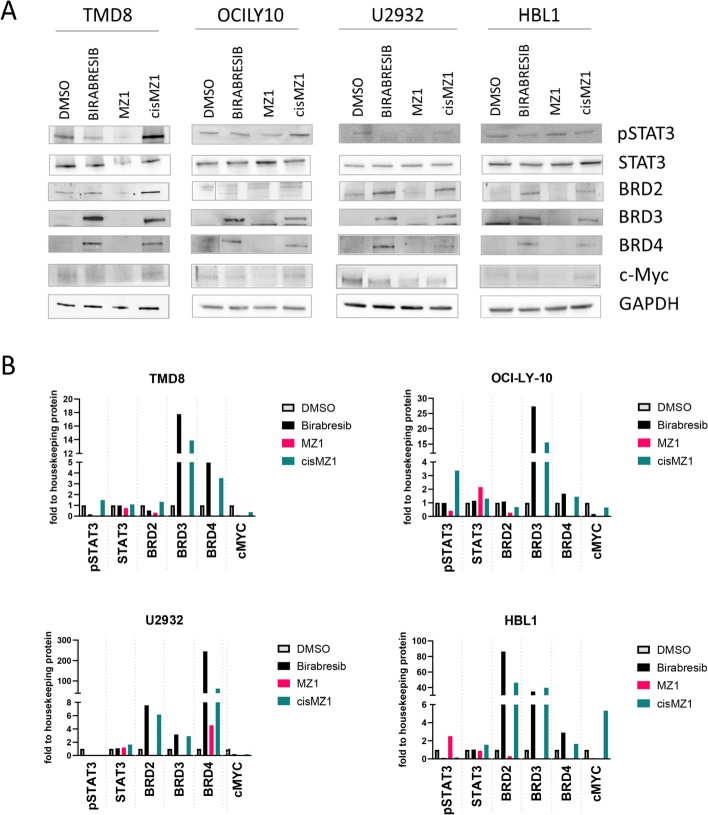
BRD proteins, MYC and pSTAT3 immunoblotting after birabresib or MZ1 treatment. Four ABC-DLBCL cell lines were treated with 500 nmol/L of DMSO, birabresib, MZ1 or cisMZ1. Protein extraction was performed after 4 h of exposure to the compound. GAPDH was used as a loading control. A. Representative immunoblottings; B. relative quantification of protein levels normalized to GAPDH

Since MZ1 presented a stronger anti-tumor activity than birabresib, we assessed its effect on proteins known to be affected by BET inhibitors. We exposed four ABC DLBCL cell lines to the two compounds for 4 h (Figure 2). MZ1 determined a strong down-regulation of its direct target BRD4 and of the other BRD proteins (BRD2 and BRD3) and abrogated MYC protein levels. Birabresib-induced MYC down-regulation was less than after MZ1 and induced an up-regulation of BRD2, BRD3, and BRD4. The MZ1 negative control epimer cisMZ1 did not cause any protein change. Based on the reported mechanism of action of BET inhibitors in ABC DLBCL [7, 9], we analyzed the effect of MZ1 on phosphorylated STAT3 (pSTAT3). We detected reduced phosphorylation in STAT3-Tyr705 after birabresib treatment, stronger with MZ1, while the protein expression level of total STAT3 was not affected by any treatment.

### The BET degrader MZ1 has *in vivo* antitumor activity in ABC DLBCL

**Figure 3.**
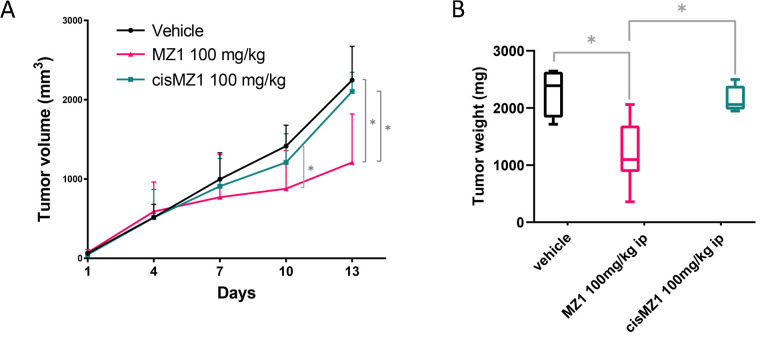
Antitumor *in vivo* activity of MZ1 in an ABC DLBCL model. Mice were treated with vehicle (i.p., 3 days ON/4 days OFF), MZ1 (100 mg/kg i.p., 3 days ON/4 days OFF), cisMZ1 (100 mg/kg, i.p., 3 days ON/4 days OFF). (A) Lines show median values per timepoint with the corresponding upper interquartile range. Y-axis, tumor volume in mm^3^; X-axis, days of treatment. (B) Tumor weight represented at autopsy illustrated as Tukey box plot showing the median and the 25–75 percentile of the treatments. ^*^ *P* < 0.05

To confirm the anti-tumor activity of MZ1 in an *in vivo* model, we engrafted the ABC DLBCL cell line TMD8 in NOD-SCID mice and we treated them with MZ1 (100 mg/kg, i.p.; 3 days ON/4 days OFF), its negative control epimer cisMZ1 (100 mg/kg, i.p.; 3 days ON/4 days OFF) or with vehicle only (Figure 3). MZ1 presented ant-tumor activity at day 10 (*P* = 0.023) and 13 (*P* = 0.014) when compared to vehicle. The cisMZ1 did not decrease the growth of the tumor cells. Tumor weight was significantly different between MZ1 (median = 1,098.4 mg, 95% CI 2,059.4–358) and vehicle (median = 2,388.7 mg, 95% CI 2,645–1,716.2; *P* = 0.012), and between MZ1 and cisMZ1 (median = 2,059.4 mg, 95% CI 2,498.3–1,945; *P* = 0.024). Treatments were well tolerated in mice, without significant signs of toxicity. Throughout treatment, mice were well-conditioned with a body condition score of body conditioning score 3 (BC3) for all groups.

### The BET degrader MZ1 causes distinct transcriptome changes compared to birabresib

**Figure 4.**
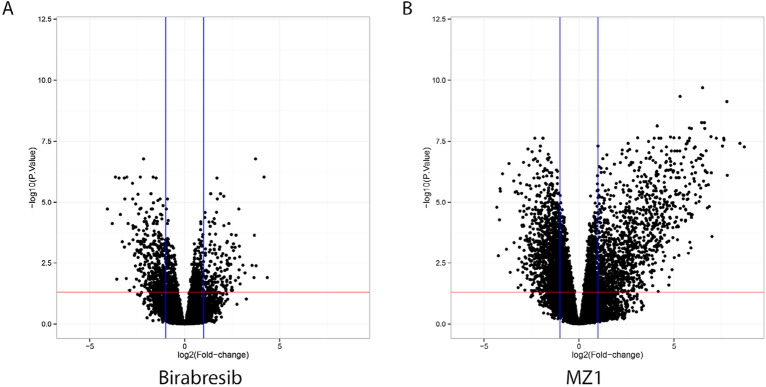
Volcano plots showing significantly upregulated (right quadrant in each plot) or down-regulated (left quadrant in each plot) transcripts after exposure to MZ1 (right panel) or birabresib (left panel) in two ABC DLBCL cell lines. Both MZ1 and birabresib were compared with DMSO. Y axis, log10-adjusted *P*-value; X axis, log2-fold changes after birabresib or MZ1 treatment

To assess the differences in transcription regulation, we exposed two ABC DLBCL cell lines (OCI-LY-10 and TMD8) to MZ1 (100 nmol/L), birabresib (100 nmol/L), or DMSO for 6 h (Figure 4, Figure S2, Table S1A-B). Considering coding genes, the number of differentially expressed transcripts was greater after MZ1 than after birabresib (MZ1 *n* = 885, birabresib *n* = 213; *P* = 0.0001, one tail Fisher’s exact test), indicating that the BET degrader MZ1 had a bigger impact on the transcriptome of ABC DLBCL than the BET inhibitor birabresib.

MYC signatures and MYC itself were downregulated by both molecules (Figure S3A, Table S2). Both MZ1 and birabresib also downregulated signatures related to interferon, JAK/STAT pathway, MYD88, NF-κB and B-cell receptor (BCR) signaling (Figure S3B, Table S2). Different to birabresib, MZ1 upregulated transcripts related to translation, ribosomal proteins, and metabolism of RNA (Table S2).

**Figure 5.**
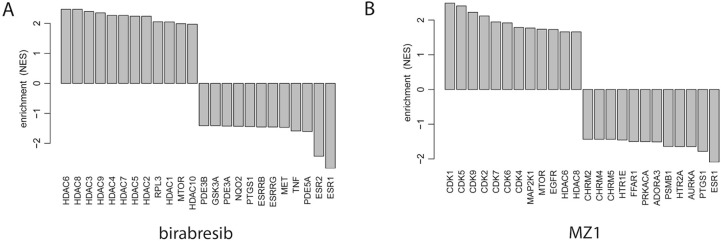
Drug connectivity enrichment plots showing the top pharmacological targets having similar or opposite enrichment in birabresib (left panel) or MZ1 (right panel) signatures obtained in two ABC DLBCL cell lines exposed to the two compounds or to DMSO, as control. RPL3: ribosomal protein L3; PDE3B: phosphodiesterase 3B; GSK3A: glycogen synthase kinase 3 alpha; NQO2: *N*-ribosyldihydronicotinamide:quinone reductase 2; PTGS1: prostaglandin-endoperoxide synthase 1; ESRRB: estrogen related receptor beta; ESRRG: estrogen related receptor gamma; MET: MET proto-oncogene, receptor tyrosine kinase; TNF: tumor necrosis factor; PDE5A: phosphodiesterase 5A; ESR2: estrogen receptor 2; MAP2K1: mitogen-activated protein kinase kinase 1; EGFR: epidermal growth factor receptor; CHRM2: cholinergic receptor muscarinic 2; HTR1E: 5-hydroxytryptamine receptor 1E; FFAR1: free fatty acid receptor 1; PRKACA: protein kinase CAMP-activated catalytic subunit alpha; ADORA3: adenosine A3 receptor; PSMB1: proteasome 20S subunit beta 1; HTR2A: 5-hydroxytryptamine receptor 2A; AURKA: aurora kinase A

The changes induced by both compounds overlapped with signatures for BET and histone deacetylase (HDAC) inhibitors and mammalian/mechanistic target of rapamycin (mTOR) inhibitors, but only the effect given by MZ1 was similar to what was reported for BET degraders such as ARV-771 and multiple cyclin-dependent kinase (CDK) inhibitors (Figure 5, Figure S4, Tables S2–S3).

While genes coding for histones were upregulated by both BET degrader and inhibitor, HEXIM P-TEFb complex subunit 1 (HEXIM1) and sestrin 3 (SESN3), two other transcripts previously reported as commonly upregulated by BET inhibitors in multiple models [15, 36, 54– 59], were upregulated only by birabresib but not by MZ1 (Table S1). As previously reported [60], the transcription factor 4 (TCF4) was downregulated by the BET inhibitor and especially by the degrader (Table S1).

## Discussions

In this study, we showed that the BET degrader MZ1 had *in vitro* and *in vivo* anti-tumor activity in ABC DLBCL cell lines. MZ1 specifically degraded BRD4, decreased the expression of MYC and the phosphorylation of STAT3. MZ1 was more potent in terms of anti-tumor activity and of modulation of the transcriptome than the BET inhibitor birabresib, which was used in parallel.

Among DLBCL, ABC DLBCL and especially its newly defined MCD/C5/MYD88 subtype are characterized by a poor outcome when patients are treated with the standard regimens [2–6]. The constitutive activation of the NF-κB and JAK/STAT3 signaling is perhaps the main feature of the lymphomas belonging to these subtypes [2–6]. Due to their strong suppression of this signaling axis, BET inhibitors have shown promising preclinical and early clinical activity in ABC DLBCL [7–12]. However, their efficacy has been limited by different factors, including an upregulation of BRD4 itself which follows the exposure to the drugs and hinders their anti-tumor activity [12, 15, 26–32, 34–37]. We showed that MZ1 induced cell death in all the ABC DLBCL cell lines tested, much more commonly than the BET-inhibitor, which had a much more limited cytotoxic capacity, in accordance with previous data [9]. The finding is in line with results obtained with other BET degraders in models derived from mantle cell lymphoma [61], and castration-resistance prostate cancer [62]. This effect is sustained by stronger effects on BRD4 than what is achieved with BET inhibitors. Indeed, while both MZ1 and birabresib downregulated MYC, BRD4 was degraded specifically by the BET degrader, as its typical effect of the E3 ligase recruitment to the proteasome. The difference between BET inhibitors and degraders is that the latter not only blocks the binding of the BET protein to histones, but it completely removes the protein, also avoiding a rebound in protein levels, often seen after BET inhibitors [35].

Similarly to what was seen for cell death, also RNA-seq experiments revealed a bigger effect for MZ1 than birabresib, but the distribution of up and down transcripts was quite similar, with the down-regulation of MYC and MYC targets, genes coding members of the interferon, JAK/STAT3 (including pSTAT3 downregulation), MYD88, NF-κB, and BCR signaling pathways. However, the comparison between transcriptional changes induced by the BET degrader MZ1 and the BET inhibitor birabresib also identified differences. The changes induced by MZ1, and not by birabresib, overlapped with the signatures reported for inhibitors of CDKs, including CDK9. CDKs are composed of a serine/threonine-catalytic domain and by a cyclin-binding domain which binds the regulatory subunit to control kinase activity and substrate specificity [63]. They have a fundamental role in cell cycle regulation, transcriptional control of cell cycle transition, regulation of RNA polymerase (RNA Pol II), proteolytic degradation, and epigenetic machine. Many CDK/cyclin complexes have a catalytic activity towards the C-terminal repeat domain of RNA Pol II. As an example, CDK9 and cyclin T belong to the positive transcription elongation factor b (P-TEFb), promoting transcription elongation. The transcriptional changes mediated by the BET degrader in ABC DLBCL cells correlate with that induced by CDK9 inhibitors, in line with results available in acute leukemias [64]. The expression profile of the BET degrader dBET6 correlates more with the changes induced by NVP-2, a potent and selective adenosine 5’-triphosphate (ATP)-competitive inhibitor of CDK9, than to the changes induced by JQ1 [64]. BET degradation does not affect CDK9 or cyclin T recruitment in the Pol II transcription elongation complex [64].

The interaction with the tumor microenvironment can sustain lymphoma cells’ growth and survival [3, 65]. Interestingly, BET inhibitors or degraders can represent a therapeutic approach to target the tumor cells both directly and via modulation of the tumor microenvironment [66]. BET degraders modulate the major histocompatibility complex class I (MHC-I) immunopeptidome inducing the presentation of peptides derived from BET proteins and other endogenous cellular proteins, which can be recognized by CD8^+^ T-cells [67]. In ABC DLBCL cells, MZ1 but not birabresib induced the expression of C-C motif chemokine ligand 3 (CCL3) and CCL4 (Table S1), two chemokines that can foster antitumor response [68–71]. CCL3 and CCL4 chemoattract the infiltration of neutrophils, macrophages, natural killer cells, and T cells to the tumor microenvironment. In the tumor-draining lymph nodes, antitumor immunity is driven by CCL3-dependent interferon gamma (IFNγ) production and CCL3-induced dendritic cells maturation [72]. In addition, *in vitro* and *in vivo* studies in Burkitt lymphoma cells suggest that CCL3 and CCL4 contribute to the rituximab-induced activation of the innate immunity system [73]. Nevertheless, some caution has to be taken into account, since the recruitment of immune cells could facilitate the survival of B-cell lymphoid neoplasms [74], and high CCL3 and CCL4 serum concentrations have been associated with an inferior outcome [75, 76]. The latter phenomenon reflects a strong BCR pathway activation in neoplastic B-cells [76] and leads to a higher sensitivity to BCR inhibitors [44, 76], which are indeed actually synergistic when combined with BET inhibition or degradation [44, 77–81].

In agreement with the literature [60], the transcription factor TCF4 was downregulated after exposure by both BET inhibitors and degraders. The gene is upregulated in ABC DLBCL when compared to GCB DLBCL [60], and indeed all but one of our cell lines, which were all derived from ABC DLBCL had indeed a gain or amplification of TCF4 locus.

HEXIM1 upregulation has been indicated as a good pharmacodynamic marker in clinical trials investigating BET inhibitors [15, 54, 55], but discordant data are available under exposure to BET degrader [61, 64]. We observed that, differently from birabresib, MZ1 did not determine HEXIM1 upregulation. Thus, in line with what was reported in T cell acute lymphoblastic leukemia cells exposed to JQ1 or to the BET degrader dBET6 [64], our data indicate that HEXIM1 levels do not appear an appropriate pharmacodynamic marker for BET degraders.

MZ1 has been shown active also in other tumor models [82–85], including in a canine DLBCL cell line [45], but one of the resistance mechanisms to PROTACs is the inactivation of the endogenous E3 ligases the compounds rely on to degrade their targets. MZ1 degrades BRD4 in colorectal cancer cell lines resistant to the BET degrader dBETi, which hijacks cereblon and not VHL [82]. Thus, compounds targeting the same protein but exploiting different E3 ligases might represent a modality to overcome resistance, and MZ1 could be implemented alongside the other BET degraders that have shown anti-lymphoma activity [61].

In conclusion, our data demonstrate the *in vitro* and *in vivo* anti-lymphoma activity of the BET degrader MZ1, characterized by the ability to induce cell death and wide effects on the transcriptome of ABC DLBCL cells, providing the rationale for its further development in this disease.

## Abbreviations

ABC: activated B-cell
AUC: area under the curve
BCR: B-cell receptor
BD: bromodomains
BET: bromodomain and extra-terminal domain
BRD2: bromodomain-containing protein 2
C5: cluster 5
CCL3: C-C motif chemokine ligand 3
CDK9: cyclin-dependent kinase 9
CI: confidence interval
DLBCL: diffuse large B cell lymphoma
DMSO: dimethyl sulfoxide
GAPDH: glyceraldehyde-3-phosphate dehydrogenase
GCB: germinal center B-cell
HDAC: histone deacetylase
HEXIM1: HEXIM P-TEFb complex subunit 1
IC_50_: half maximal inhibitory concentration
JAK: Janus tyrosine kinase
mTOR: mammalian/mechanistic target of rapamycin
MYD88: myeloid differentiation factor 88
NF-κB: nuclear factor kappa B
NOD-SCID: NOD.CB17-Prkdcscid/NCrHsd
PBS: phosphate buffered saline
pSTAT3: phosphorylated signal transducer and activator of transcription 3
STAT3: signal transducer and activator of transcription 3
TCF4: transcription factor 4

## Supplementary materials

The supplementary Figures for this article are available at: https://www.explorationpub.com/uploads/Article/file/100265_sup_1.pdf. The supplementary Tables for this article are available at: https://www.explorationpub.com/uploads/Article/file/100265_sup_2.xlsx.

## Declarations

### Availability of data and materials

All transcriptome data are available at GEO (http://www.ncbi.nlm.nih.gov/geo).

### Copyright

© The Author(s) 2021.

## References

[1] Armitage JO Gascoyne RD Lunning MA Cavalli F. Non-Hodgkin lymphoma. Lancet. 2017;390:298–310. 10.1016/S0140-6736(16)32407-2 28153383

[2] Pasqualucci L Dalla-Favera R. Genetics of diffuse large B-cell lymphoma. Blood. 2018;131:2307–19. 10.1182/blood-2017-11-764332 29666115 PMC5969374

[3] Miao Y Medeiros LJ Li Y Li J Young KH. Genetic alterations and their clinical implications in DLBCL. Nat Rev Clin Oncol. 2019;16:634–52. 10.1038/s41571-019-0225-1 31127191

[4] Wright GW Huang DW Phelan JD Coulibaly ZA Roulland S Young RM A probabilistic classification tool for genetic subtypes of diffuse large B cell lymphoma with therapeutic implications. Cancer Cell. 2020;37:551–68.e14. 10.1016/j.ccell.2020.03.015 32289277 PMC8459709

[5] Cascione L Aresu L Baudis M Bertoni F. DNA copy number changes in diffuse large B cell lymphomas. Front Oncol. 2020;10:584095. 10.3389/fonc.2020.584095 33344238 PMC7740002

[6] Chapuy B Stewart C Dunford AJ Kim J Kamburov A Redd RA Molecular subtypes of diffuse large B cell lymphoma are associated with distinct pathogenic mechanisms and outcomes. Nat Med. 2018;24:679–90. 10.1038/s41591-018-0016-8 29713087 PMC6613387

[7] Ceribelli M Kelly PN Shaffer AL Wright GW Xiao W Yang Y Blockade of oncogenic IκB kinase activity in diffuse large B-cell lymphoma by bromodomain and extraterminal domain protein inhibitors. Proc Natl Acad Sci U S A. 2014;111:11365–70. 10.1073/pnas.1411701111 25049379 PMC4128109

[8] Chapuy B McKeown MR Lin CY Monti S Roemer MG Qi J Discovery and characterization of super-enhancer-associated dependencies in diffuse large B cell lymphoma. Cancer Cell. 2013;24:777–90. 10.1016/j.ccr.2013.11.003 24332044 PMC4018722

[9] Boi M Gaudio E Bonetti P Kwee I Bernasconi E Tarantelli C The BET bromodomain inhibitor OTX015 affects pathogenetic pathways in preclinical B-cell tumor models and synergizes with targeted drugs. Clin Cancer Res. 2015;21:1628–38. 10.1158/1078-0432.CCR-14-1561 25623213

[10] Bernasconi E Gaudio E Lejeune P Tarantelli C Cascione L Kwee I Preclinical evaluation of the BET bromodomain inhibitor BAY 1238097 for the treatment of lymphoma. Br J Haematol. 2017;178:936–48. 10.1111/bjh.14803 28653353

[11] Blum KA Abramson J Maris M Flinn I Goy A Mertz J A phase I study of CPI-0610, a bromodomain and extra terminal protein (BET) inhibitor in patients with relapsed or refractory lymphoma. Ann Oncol. 2018;29:iii7–9. 10.1093/annonc/mdy048

[12] Amorim S Stathis A Gleeson M Iyengar S Magarotto V Leleu X Bromodomain inhibitor OTX015 in patients with lymphoma or multiple myeloma: a dose-escalation, open-label, pharmacokinetic, phase 1 study. Lancet Haematol. 2016;3:e196–204. 10.1016/S2352-3026(16)00021-1 27063978

[13] Stathis A Bertoni F. BET proteins as targets for anticancer treatment. Cancer Discov. 2018;8:24–36. 10.1158/2159-8290.CD-17-0605 29263030

[14] Donati B Lorenzini E Ciarrocchi A. BRD4 and cancer: going beyond transcriptional regulation. Mol Cancer.2018;17:164. 10.1186/s12943-018-0915-9 30466442 PMC6251205

[15] Spriano F Stathis A Bertoni F. Targeting BET bromodomain proteins in cancer: the example of lymphomas. Pharmacol Ther. 2020;215:107631. 10.1016/j.pharmthera.2020.107631 32693114

[16] Lambert JP Picaud S Fujisawa T Hou H Savitsky P Uusküla-Reimand L Interactome rewiring following pharmacological targeting of BET bromodomains. Mol Cell. 2019;73:621–38.e17. 10.1016/j.molcel.2018.11.006 30554943 PMC6375729

[17] Andrieu GP Denis GV. BET proteins exhibit transcriptional and functional opposition in the epithelial-to-mesenchymal transition. Mol Cancer Res. 2018;16:580–6. 10.1158/1541-7786.MCR-17-0568 29437854 PMC5882530

[18] Belkina AC Denis GV. BET domain co-regulators in obesity, inflammation and cancer. Nat Rev Cancer. 2012;12:465–77. 10.1038/nrc3256 22722403 PMC3934568

[19] Deeney JT Belkina AC Shirihai OS Corkey BE Denis GV. BET bromodomain proteins Brd2, Brd3 and Brd4 selectively regulate metabolic pathways in the pancreatic β-cell. PLoS One. 2016;11:e0151329. 10.1371/journal.pone.0151329 27008626 PMC4805167

[20] Filippakopoulos P Knapp S. Targeting bromodomains: epigenetic readers of lysine acetylation. Nat Rev Drug Discov. 2014;13:337–56. 10.1038/nrd4286 24751816

[21] Delmore JE Issa GC Lemieux ME Rahl PB Shi J Jacobs HM BET bromodomain inhibition as a therapeutic strategy to target c-Myc. Cell. 2011;146:904–17. 10.1016/j.cell.2011.08.017 21889194 PMC3187920

[22] Phelan JD Young RM Webster DE Roulland S Wright GW Kasbekar M A multiprotein supercomplex controlling oncogenic signalling in lymphoma. Nature. 2018;560:387–91. 10.1038/s41586-018-0290-0 29925955 PMC6201842

[23] Zou Z Huang B Wu X Zhang H Qi J Bradner J Brd4 maintains constitutively active NF-κB in cancer cells by binding to acetylated RelA. Oncogene. 2014;33:2395–404. 10.1038/onc.2013.179 23686307 PMC3913736

[24] Greenwald RJ Tumang JR Sinha A Currier N Cardiff RD Rothstein TL E mu-BRD2 transgenic mice develop B-cell lymphoma and leukemia. Blood. 2004;103:1475–84. 10.1182/blood-2003-06-2116 14563639 PMC2825482

[25] Belkina AC Blanton WP Nikolajczyk BS Denis GV. The double bromodomain protein Brd2 promotes B cell expansion and mitogenesis. J Leukoc Biol. 2014;95:451–60. 10.1189/jlb.1112588 24319289 PMC3923082

[26] Berthon C Raffoux E Thomas X Vey N Gomez-Roca C Yee K Bromodomain inhibitor OTX015 in patients with acute leukaemia: a dose-escalation, phase 1 study. Lancet Haematol. 2016;3:e186–95. 10.1016/S2352-3026(15)00247-1 27063977

[27] Lewin J Soria JC Stathis A Delord JP Peters S Awada A Phase Ib trial with birabresib, a small- molecule inhibitor of bromodomain and extraterminal proteins, in patients with selected advanced solid tumors. J Clin Oncol. 2018;36:3007–14. 10.1200/JCO.2018.78.2292 29733771

[28] Piha-Paul SA Sachdev JC Barve M LoRusso P Szmulewitz R Patel SP First-in-human study of mivebresib (ABBV-075), an oral pan-inhibitor of bromodomain and extra terminal proteins, in patients with relapsed/refractory solid tumors. Clin Cancer Res. 2019;25:6309–19. 10.1158/1078-0432.CCR-19-0578 31420359

[29] Postel-Vinay S Herbschleb K Massard C Woodcock V Soria JC Walter AO First-in-human phase I study of the bromodomain and extraterminal motif inhibitor BAY 1238097: emerging pharmacokinetic/ pharmacodynamic relationship and early termination due to unexpected toxicity. Eur J Cancer. 2019;109:103–10. 10.1016/j.ejca.2018.12.020 30711772

[30] Falchook G Rosen S LoRusso P Watts J Gupta S Coombs CC Development of 2 bromodomain and extraterminal inhibitors with distinct pharmacokinetic and pharmacodynamic profiles for the treatment of advanced malignancies. Clin Cancer Res. 2020;26:1247–57. 10.1158/1078-0432.CCR-18-4071 31527168 PMC7528620

[31] Moreno V Sepulveda JM Vieito M Hernández-Guerrero T Doger B Saavedra O Phase I study of CC-90010, a reversible, oral BET inhibitor in patients with advanced solid tumors and relapsed/ refractory non-Hodgkin’s lymphoma. Ann Oncol. 2020;31:780–8. 10.1016/j.annonc.2020.03.294 32240793

[32] Piha-Paul SA Hann CL French CA Cousin S Braña I Cassier PA Phase 1 study of molibresib (GSK525762), a bromodomain and extra-terminal domain protein inhibitor, in NUT carcinoma and other solid tumors. JNCI Cancer Spectr. 2019;4:pkz093. 10.1093/jncics/pkz093 32328561 PMC7165800

[33] Shapiro GI LoRusso P Dowlati A T Do K Jacobson CA Vaishampayan U A phase 1 study of RO6870810, a novel bromodomain and extra-terminal protein inhibitor, in patients with NUT carcinoma, other solid tumours, or diffuse large B-cell lymphoma. Br J Cancer. 2021;124:744–53. 10.1038/s41416-020-01180-1 33311588 PMC7884382

[34] Shimamura T Chen Z Soucheray M Carretero J Kikuchi E Tchaicha JH Efficacy of BET bromodomain inhibition in Kras-mutant non-small cell lung cancer. Clin Cancer Res. 2013;19:6183–92. 10.1158/1078-0432.CCR-12-3904 24045185 PMC3838895

[35] Lu J Qian Y Altieri M Dong H Wang J Raina K Hijacking the E3 ubiquitin ligase cereblon to efficiently target BRD4. Chem Biol. 2015;22:755–63. 10.1016/j.chembiol.2015.05.009 26051217 PMC4475452

[36] Astorgues-Xerri L Vázquez R Odore E Rezai K Kahatt C Mackenzie S Insights into the cellular pharmacological properties of the BET-inhibitor OTX015/MK-8628 (birabresib), alone and in combination, in leukemia models. Leuk Lymphoma. 2019;60:3067–70. 10.1080/10428194.2019.1617860 31204545

[37] Vázquez R Riveiro ME Astorgues-Xerri L Odore E Rezai K Erba E The bromodomain inhibitor OTX015 (MK-8628) exerts anti-tumor activity in triple-negative breast cancer models as single agent and in combination with everolimus. Oncotarget. 2017;8:7598–613. 10.18632/oncotarget.13814 27935867 PMC5352346

[38] Raina K Crews CM. Chemical inducers of targeted protein degradation. J Biol Chem. 2010;285:11057–60. 10.1074/jbc.R109.078105 20147751 PMC2856979

[39] Dale B Cheng M Park KS Kaniskan HÜ Xiong Y Jin J. Advancing targeted protein degradation for cancer therapy. Nat Rev Cancer. 2021;21:638–54. 10.1038/s41568-021-00365-x 34131295 PMC8463487

[40] Troup RI Fallan C Baud MGJ. Current strategies for the design of PROTAC linkers: a critical review. Explor Target Antitumor Ther. 2020;1:273–312. 10.37349/etat.2020.00018 PMC9400730 36046485

[41] Duan Y Guan Y Qin W Zhai X Yu B Liu H. Targeting Brd4 for cancer therapy: inhibitors and degraders. Medchemcomm. 2018;9:1779–802. 10.1039/C8MD00198G 30542529 PMC6238758

[42] Zengerle M Chan KH Ciulli A. Selective small molecule induced degradation of the BET bromodomain protein BRD4. ACS Chem Biol. 2015;10:1770–7. 10.1021/acschembio.5b00216 26035625 PMC4548256

[43] Gaudio E Tarantelli C Spriano F Guidetti F Sartori G Bordone R Targeting CD205 with the antibody drug conjugate MEN1309/OBT076 is an active new therapeutic strategy in lymphoma models. Haematologica. 2020;105:2584–91. 10.3324/haematol.2019.227215 33131247 PMC7604571

[44] Tarantelli C Gaudio E Arribas AJ Kwee I Hillmann P Rinaldi A PQR309 is a novel dual PI3K/mTOR inhibitor with preclinical antitumor activity in lymphomas as a single agent and in combination therapy. Clin Cancer Res. 2018;24:120–9. 10.1158/1078-0432.CCR-17-1041 29066507

[45] Aresu L Ferraresso S Marconato L Cascione L Napoli S Gaudio E New molecular and therapeutic insights into canine diffuse large B-cell lymphoma elucidates the role of the dog as a model for human disease. Haematologica. 2019;104:e256–9. 10.3324/haematol.2018.207027 30545928 PMC6545862

[46] Ullman-Culleré MH Foltz CJ. Body condition scoring: a rapid and accurate method for assessing health status in mice. Lab Anim Sci. 1999;49:319–23. 10403450

[47] Spriano F Chung EYL Gaudio E Tarantelli C Cascione L Napoli S The ETS inhibitors YK-4-279 and TK-216 are novel antilymphoma agents. Clin Cancer Res. 2019;25:5167–76. 10.1158/1078-0432.CCR-18-2718 31182435

[48] Cascione L Rinaldi A Bruscaggin A Tarantelli C Arribas AJ Kwee I Novel insights into the genetics and epigenetics of MALT lymphoma unveiled by next generation sequencing analyses. Haematologica. 2019;104:e558–61. 10.3324/haematol.2018.214957 31018978 PMC6959164

[49] Subramanian A Tamayo P Mootha VK Mukherjee S Ebert BL Gillette MA Gene set enrichment analysis: a knowledge-based approach for interpreting genome-wide expression profiles. Proc Natl Acad Sci U S A. 2005;102:15545–50. 10.1073/pnas.0506580102 16199517 PMC1239896

[50] Shaffer AL Wright G Yang L Powell J Ngo V Lamy L A library of gene expression signatures to illuminate normal and pathological lymphoid biology. Immunol Rev. 2006;210:67–85. 10.1111/j.0105-2896.2006.00373.x 16623765

[51] Akhmedov M Martinelli A Geiger R Kwee I. Omics playground: a comprehensive self-service platform for visualization, analytics and exploration of Big Omics Data. NAR Genom Bioinform. 2019;2:lqz019. 10.1093/nargab/lqz019 33575569 PMC7671354

[52] Subramanian A Narayan R Corsello SM Peck DD Natoli TE Lu X A next generation connectivity map: L1000 platform and the first 1,000,000 profiles. Cell. 2017;171:1437–52.e17. 10.1016/j.cell.2017.10.049 29195078 PMC5990023

[53] Spriano F Gaudio E Cascione L Tarantelli C Melle F Motta G Antitumor activity of the dual BET and CBP/EP300 inhibitor NEO2734. Blood Adv. 2020;4:4124–35. 10.1182/bloodadvances.2020001879 32882003 PMC7479962

[54] Yeh TC O’Connor G Petteruti P Dulak A Hattersley M Barrett JC Identification of CCR2 and CD180 as robust pharmacodynamic tumor and blood biomarkers for clinical use with BRD4/BET inhibitors. Clin Cancer Res. 2017;23:1025–35. 10.1158/1078-0432.CCR-16-1658 28073847

[55] Lin X Huang X Uziel T Hessler P Albert DH Roberts-Rapp LA HEXIM1 as a robust pharmacodynamic marker for monitoring target engagement of BET family bromodomain inhibitors in tumors and surrogate tissues. Mol Cancer Ther. 2017;16:388–96. 10.1158/1535-7163.MCT-16-0475 27903752

[56] Mertz JA Conery AR Bryant BM Sandy P Balasubramanian S Mele DA Targeting MYC dependence in cancer by inhibiting BET bromodomains. Proc Natl Acad Sci U S A. 2011;108:16669–74. 10.1073/pnas.1108190108 21949397 PMC3189078

[57] Ott CJ Kopp N Bird L Paranal RM Qi J Bowman T BET bromodomain inhibition targets both c-Myc and IL7R in high-risk acute lymphoblastic leukemia. Blood. 2012;120:2843–52. 10.1182/blood-2012-02-413021 22904298 PMC3466965

[58] Berenguer-Daizé C Astorgues-Xerri L Odore E Cayol M Cvitkovic E Noel K OTX015 (MK-8628), a novel BET inhibitor, displays *in vitro* and *in vivo* antitumor effects alone and in combination with conventional therapies in glioblastoma models. Int J Cancer. 2016;139:2047–55. 10.1002/ijc.30256 27388964

[59] Riveiro ME Kwee I Astorgues-Xerri L Bekradda M Vazquez R Rinaldi A Abstract 3530: gene expression profile of OTX015, a BET bromodomain inhibitor, in preclinical models of non-small-cell lung cancer (NSCLC) and small-cell lung cancer (SCLC) models. Cancer Res. 2015;75:3530. 10.1158/1538-7445.AM2015-3530

[60] Jain N Hartert K Tadros S Fiskus W Havranek O Ma MCJ Targetable genetic alterations of TCF4 (E2-2) drive immunoglobulin expression in diffuse large B cell lymphoma. Sci Transl Med. 2019;11:eaav5599. 10.1126/scitranslmed.aav5599 31217338 PMC6724184

[61] Sun B Fiskus W Qian Y Rajapakshe K Raina K Coleman KG BET protein proteolysis targeting chimera (PROTAC) exerts potent lethal activity against mantle cell lymphoma cells. Leukemia. 2018;32:343–52. 10.1038/leu.2017.207 28663582 PMC12856936

[62] Raina K Lu J Qian Y Altieri M Gordon D Rossi AM PROTAC-induced BET protein degradation as a therapy for castration-resistant prostate cancer. Proc Natl Acad Sci U S A. 2016;113:7124–9. 10.1073/pnas.1521738113 27274052 PMC4932933

[63] Lim S Kaldis P. Cdks, cyclins and CKIs: roles beyond cell cycle regulation. Development. 2013;140:3079–93. 10.1242/dev.091744 23861057

[64] Winter GE Mayer A Buckley DL Erb MA Roderick JE Vittori S BET bromodomain proteins function as master transcription elongation factors independent of CDK9 pecruitment. Mol Cell. 2017;67:5–18.e19. 10.1016/j.molcel.2017.06.004 28673542 PMC5663500

[65] Pizzi M Boi M Bertoni F Inghirami G. Emerging therapies provide new opportunities to reshape the multifaceted interactions between the immune system and lymphoma cells. Leukemia. 2016;30:1805–15. 10.1038/leu.2016.161 27389058

[66] Aspeslagh S Morel D Soria JC Postel-Vinay S. Epigenetic modifiers as new immunomodulatory therapies in solid tumours. Ann Oncol. 2018;29:812–24. 10.1093/annonc/mdy050 29432557

[67] Jensen SM Potts GK Ready DB Patterson MJ. Specific MHC-I peptides are induced using PROTACs. Front Immunol. 2018;9:2697. 10.3389/fimmu.2018.02697 30524438 PMC6262898

[68] Crittenden M Gough M Harrington K Olivier K Thompson J Vile RG. Expression of inflammatory chemokines combined with local tumor destruction enhances tumor regression and long-term immunity. Cancer Res. 2003;63:5505–12. 14500387

[69] Yang X Lu P Fujii C Nakamoto Y Gao JL Kaneko S Essential contribution of a chemokine, CCL3, and its receptor, CCR1, to hepatocellular carcinoma progression. Int J Cancer. 2006;118:1869–76. 10.1002/ijc.21596 16284949

[70] Luo X Yu Y Liang A Xie Y Liu S Guo J Intratumoral expression of MIP-1beta induces antitumor responses in a pre-established tumor model through chemoattracting T cells and NK cells. Cell Mol Immunol. 2004;1:199–204. 16219168

[71] Zibert A Balzer S Souquet M Quang TH Paris-Scholz C Roskrow M CCL3/MIP-1alpha is a potent immunostimulator when coexpressed with interleukin-2 or granulocyte-macrophage colony-stimulating factor in a leukemia/lymphoma vaccine. Hum Gene Ther. 2004;15:21–34. 10.1089/10430340460732436 14965375

[72] Allen F Rauhe P Askew D Tong AA Nthale J Eid S CCL3 enhances antitumor immune priming in the lymph node via IFNγ with dependency on natural killer cells. Front Immunol. 2017;8:1390. 10.3389/fimmu.2017.01390 29109732 PMC5660298

[73] Cittera E Leidi M Buracchi C Pasqualini F Sozzani S Vecchi A The CCL3 family of chemokines and innate immunity cooperate *in vivo* in the eradication of an established lymphoma xenograft by rituximab. J Immunol. 2007;178:6616–23. 10.4049/jimmunol.178.10.6616 17475893

[74] Zucchetto A Tripodo C Benedetti D Deaglio S Gaidano G Del Poeta G Monocytes/macrophages but not T lymphocytes are the major targets of the CCL3/CCL4 chemokines produced by CD38^+^CD49d^+^ chronic lymphocytic leukaemia cells. Br J Haematol. 2010;150:111–3. 10.1111/j.1365-2141.2010.08152.x 20346010

[75] Sivina M Hartmann E Kipps TJ Rassenti L Krupnik D Lerner S CCL3 (MIP-1α) plasma levels and the risk for disease progression in chronic lymphocytic leukemia. Blood. 2011;117:1662–9. 10.1182/blood-2010-09-307249 21115978 PMC3318778

[76] Takahashi K Sivina M Hoellenriegel J Oki Y Hagemeister FB Fayad L CCL3 and CCL4 are biomarkers for B cell receptor pathway activation and prognostic serum markers in diffuse large B cell lymphoma. Br J Haematol. 2015;171:726–35. 10.1111/bjh.13659 26358140 PMC4715651

[77] Tinsley S Meja K Shepherd C Khwaja A. Synergistic induction of cell death in haematological malignancies by combined phosphoinositide-3-kinase and BET bromodomain inhibition. Br J Haematol. 2015;170:275–8. 10.1111/bjh.13283 25640480

[78] Stubbs M Maduskuie T Burn T Diamond-Fosbenner S Falahatpisheh N Volgina A Abstract 5071: preclinical characterization of the potent and selective BET inhibitor INCB057643 in models of hematologic malignancies. Cancer Res. 2017;77:5071. 10.1158/1538-7445.AM2017-5071

[79] Meadows S Yahiaoui A Sorensen R Cui ZH Brockett R Keegan KS Evaluation of idelalisib with B-cell receptor or orthogonal pathway inhibitors in diffuse large B-Cell lymphoma cell lines *in vitro* and *in vivo*. Blood. 2016;128:1845. 10.1182/blood.V128.22.1845.184527531676

[80] Kim E Ten Hacken E Sivina M Clarke A Thompson PA Jain N The BET inhibitor GS-5829 targets chronic lymphocytic leukemia cells and their supportive microenvironment. Leukemia. 2020;34:1588–98. 10.1038/s41375-019-0682-7 31862959 PMC7272263

[81] Derenzini E Mondello P Erazo T Portelinha A Liu Y Scallion M BET inhibition-induced GSK3β feedback enhances lymphoma vulnerability to PI3K inhibitors. Cell Rep. 2018;24:2155–66. 10.1016/j.celrep.2018.07.055 30134175 PMC7456333

[82] Otto C Schmidt S Kastner C Denk S Kettler J Müller N Targeting bromodomain-containing protein 4 (BRD4) inhibits MYC expression in colorectal cancer cells. Neoplasia. 2019;21:1110–20. 10.1016/j.neo.2019.10.003 31734632 PMC6888720

[83] Noblejas-López MDM Nieto-Jimenez C Burgos M Gómez-Juárez M Montero JC Esparís-Ogando A Activity of BET-proteolysis targeting chimeric (PROTAC) compounds in triple negative breast cancer. J Exp Clin Cancer Res. 2019;38:383. 10.1186/s13046-019-1387-5 31470872 PMC6717344

[84] Shafran JS Andrieu GP Györffy B Denis GV. BRD4 regulates metastatic potential of castration-resistant prostate cancer through AHNAK. Mol Cancer Res. 2019;17:1627–38. 10.1158/1541-7786.MCR-18-1279 31110158 PMC6677600

[85] Chan KH Zengerle M Testa A Ciulli A. Impact of target warhead and linkage vector on inducing protein degradation: comparison of bromodomain and extra-terminal (BET) degraders derived from triazolodiazepine (JQ1) and tetrahydroquinoline (I-BET726) BET inhibitor scaffolds. J Med Chem. 2018;61:504–13. 10.1021/acs.jmedchem.6b01912 28595007 PMC5788402

