# Supplementary material for: The bromodomain and extra-terminal domain degrader MZ1 exhibits preclinical anti-tumoral activity in diffuse large B-cell lymphoma of the activated B cell-like type
Source: Explor Target Antitumor Ther. 2021 Dec 31;2(6):586–601. doi: 10.37349/etat.2021.00065 (PMC9400774; doi:10.37349/etat.2021.00065)
Supplement: Supplementary file 1 [file etat-02-100265-s001.pdf]

The bromodomain and extra-terminal domain degrader MZ1 exhibits preclinical anti-tumoral activity in diffuse large B-cell lymphoma of the activated B cell-like type

Chiara Tarantelli[1], Eleonora Cannas[1], Hillarie Ekeh[1], Carmelo Moscatello[1,2], Eugenio Gaudio[1], Luciano Cascione[1,3], Sara Napoli[1], Cesare Rech[1], Andrea Testa[4], Chiara Maniaci[4], Andrea Rinaldi[1], Emanuele Zucca[1,5], Anastasios Stathis[5,6], Alessio Ciulli[4], Francesco Bertoni[1,5*]

[1]Institute of Oncology Research, Faculty of Biomedical Sciences, USI, 6500 Bellinzona, Switzerland

[2]Department of Medical, Oral and Biotechnological Sciences, 'G. d'Annunzio' University of Chieti-Pescara, I-66100 Chieti, Italy

[3]SIB Swiss Institute of Bioinformatics, Lausanne, Switzerland

[4]Division of Biological Chemistry and Drug Discovery, School of Life Sciences, University of Dundee, Dundee, DD1 5EH, Scotland, UK

[5]Oncology Institute of Southern Switzerland, Ente Ospedaliero Cantonale, Bellinzona, Switzerland

[6]Faculty of Biomedical Sciences, USI, 6900 Lugano, Switzerland

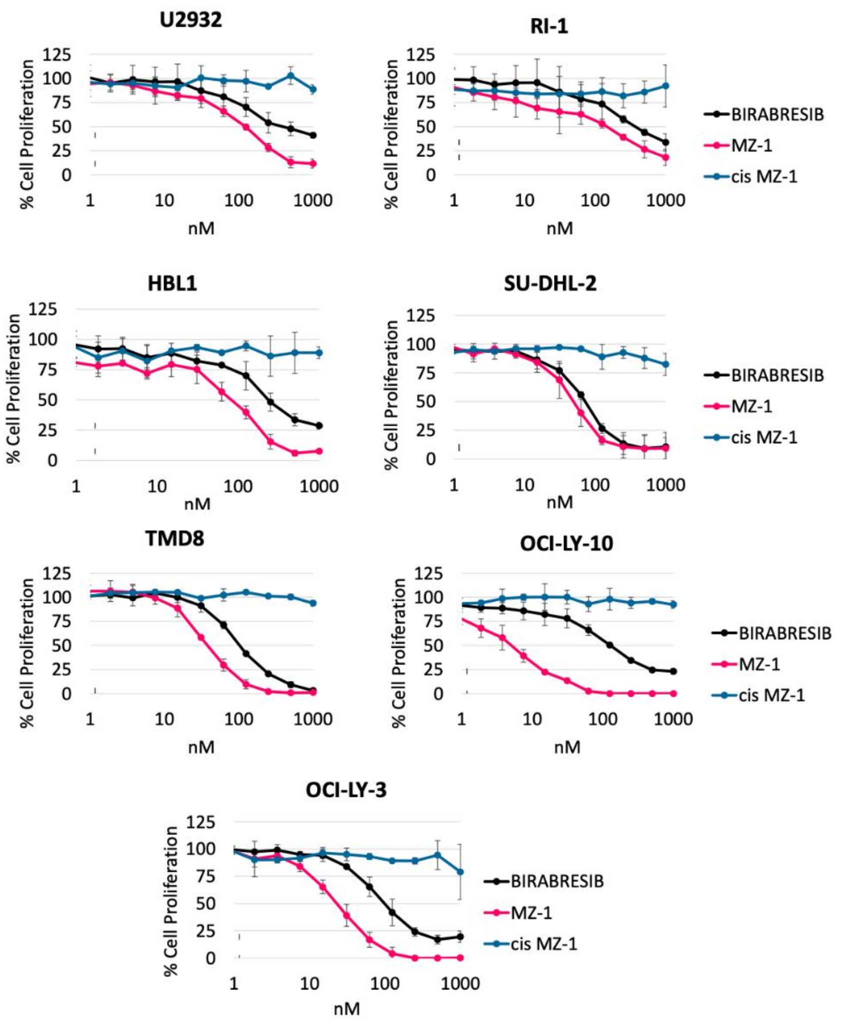

Figure S1. Drug-response curves of ABC-DLBCL cell lines to birabresib, MZ1 and cisMZ1. MTT assays of seven ABC-DLBCL cell lines exposed to birabresib, MZ1 and cisMZ1 for 72 hours. Untreated cells were used as control. Experiments were performed in duplicate.

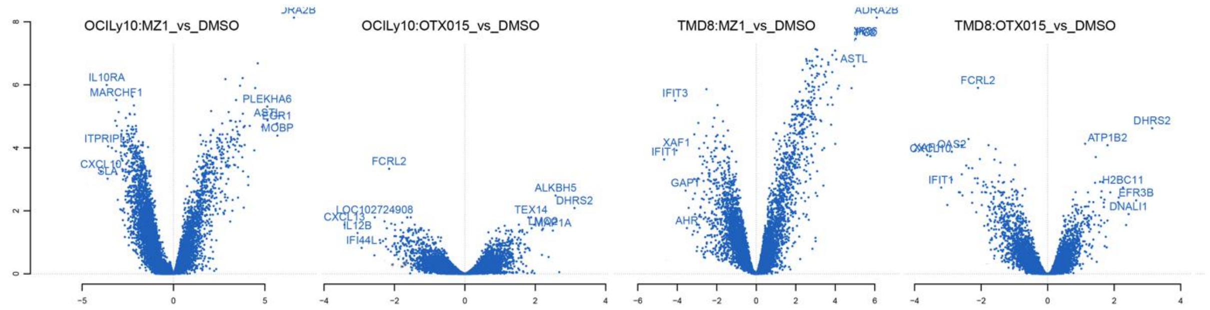

Figure S2. Volcano plots showing significantly upregulated (right quadrant in each plot) or down-regulated (left quadrant in each plot) transcripts after exposure to MZ1 or birabresib (OTX015) in OCI-LY-10 and TMD8 ABC DLBCL cell lines. Both MZ1 and birabresib were compared with DMSO. Y axis, log10-adjusted p-value; X axis, log2-fold changes after birabresib or MZ1 treatment vs DMSO

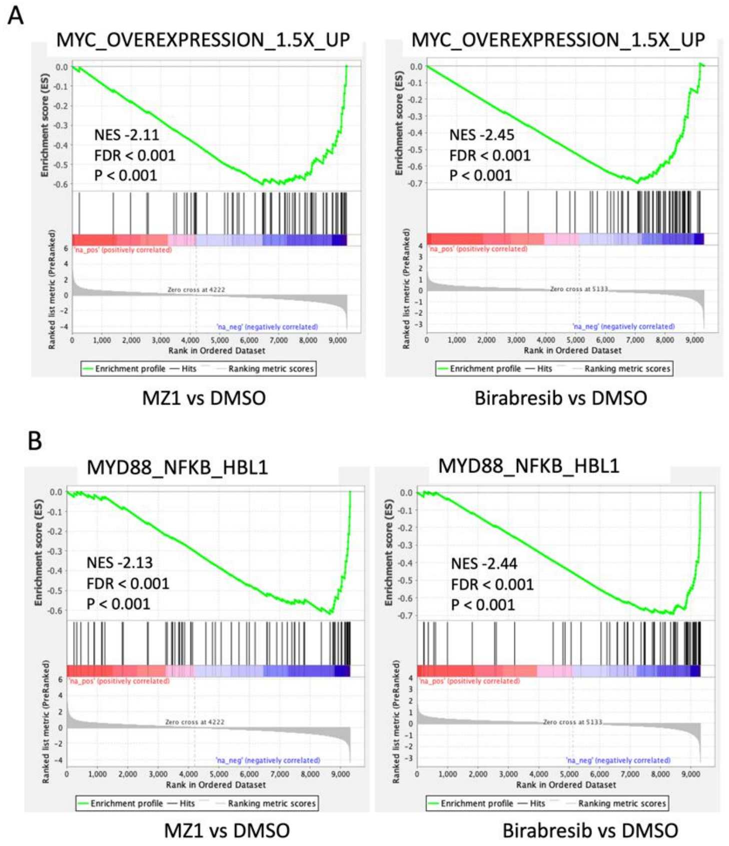

Figure S3. Enrichment of MYC and NF-kB signatures in transcripts down-regulated in the ABC DLBCL cell line exposed to MZ1 or birabresib. In each GSEA plot: Green line, enrichment score; bars in the middle portion of the plots show where the members of the gene set appear in the ranked list of genes. Positive or negative ranking metric indicate correlation or inverse correlation with the profile, respectively. FDR, false discovery rate; NES, normalized enrichment score

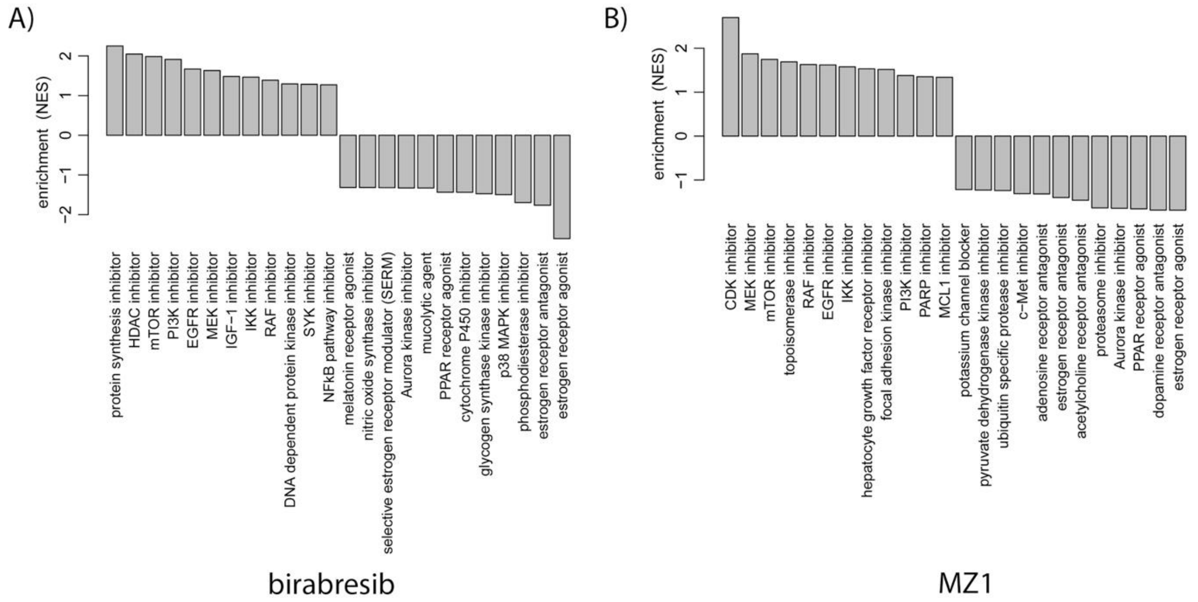

Figure S4. Drug connectivity enrichment plots showing the top pharmacological targets having similar or opposite enrichment in birabresib (left panel) or MZ1 (right panel) signatures obtained in two ABC DLBCL cell lines exposed to the two compounds or to DMSO, as control

Supplementary Tables legends

Table S1. Gene expression profiling of OCI-LY-10 cell line exposed to the BET inhibitor birabresib, BET-PROTAC inhibitor MZ1 or to DMSO. Results of limma test between DMSO and MZ1 (A) or between DMSO and birabresib (B) treated cell line

Table S2. GSEA results after MZ1 or birabresib exposure in the ABC DLBCL cell line OCI-LY-10. GSEA functional annotation of the MZ1- (A) or birabresib- (B) induced changes in up- and down-regulated genes. $P< 0.05$ and FDR values < 0.1

Table S3. Drug-connectivity enrichment table for most similar drugs compared to MZ1 or birabresib treatment. Mechanism-of-action table showing the top most frequent drugs having similar or opposite enrichment between L1000 database and MZ1 vs DMSO (A) or birabresib vs DMSO (B) signature
